# Bio-Inspired Surface Modification of Magnetite Nanoparticles with Dopamine Conjugates

**DOI:** 10.3390/nano12132230

**Published:** 2022-06-29

**Authors:** Alexander Volov, Liubov Shkodenko, Elena Koshel, Andrey S. Drozdov

**Affiliations:** 1 Department of Chemistry, Moscow State University, 119234 Moscow, Russia; volovalexander@gmail.com; 2 SCAMT Institute, ITMO University, 191002 Saint Petersburg, Russia; shkodenko@scamt-itmo.ru (L.S.); koshel@scamt-itmo.ru (E.K.); 3 Laboratory of Nanobiotechnologies, Moscow Institute of Physics and Technology, 141701 Dolgoprudny, Moscow Region, Russia

**Keywords:** magnetite, dopamine, surface modification, hybrid materials, antibacterial agents

## Abstract

Organically-coated nanomaterials are intensively studied and find numerous applications in a wide range of areas from optics to biomedicine. One of the recent trends in material science is the application of bio-mimetic polydopamine coatings that can be produced on a variety of substrates in a cost-efficient way under mild conditions. Such coatings not only modify the biocompatibility of the material but also add functional amino groups to the surface that can be further modified by classic conjugation techniques. Here we show an alternative strategy for substrates modification using dopamine conjugates instead of native dopamine. Compared to the classic scheme, the proposed strategy allows separation of the “organic” and “colloidal” stages, and simplified identification and purification steps. Modification with pre-modified dopamine made it possible to achieve high loading capacities with active components up to 10.5% wt. A series of organo-inorganic hybrids were synthesized and their bioactivity was analyzed.

## 1. Introduction

Surface properties and surface functionality play a very important role in the operational characteristics of nanomaterials. These aspects are becoming even more important when it comes to bio-applied materials due to the complexity and versatility of the interactions on the biointerfaces [1,2,3,4]. In recent decades, a large number of methods based on physical or chemical approaches have been developed for surface modification depending on proposed applications [5,6,7,8,9]. In the laboratory, the day-to-day practice of most researchers relies on chemical methods due to their availability and versatility. Silanization, polyelectrolyte layer-by-layer adsorption, graft polymerization, or click-chemistry are now widely used for surface modification and the creation of functional materials. At the same time, it could be concluded that current trends are aimed at simplification and universalization of the approaches and softening of reaction conditions.

In 2007 Lee et al. described a universal coating strategy inspired by the way mussels attach to various surfaces [10]. Proteomic analysis has discovered that the attachment of mussels is mediated through the adhesive foot protein, Mefp-5 (Mytilus edulis foot protein-5) which is rich in 3,4-dihydroxy-L-phenylalanine (DOPA) and lysine that bears catechol- and amino-groups, respectively. It was discovered that these motifs are strongly interacting with each other and other materials through hydrogen bonds, and electrostatic and hydrophobic interactions ensure strong attachment to almost all types of substrates, both organic and inorganic, including super-hydrophobic ones. This discovery led to the development of so-called polydopamine (PDA) coatings which are readily formed in mild conditions through oxidative cyclization and polymerization of dopamine by soluted oxygen [11,12,13,14,15]. Due to relative cheapness, biocompatibility, and synthetic availability, bio-inspired coatings have found application in various fields of science, such as cell patterning, theranostics, tissue engineering, photocatalysis, and Li-ion batteries [15,16,17]. Another important feature is that such self-forming coatings bearing amino groups can be further modified by typical chemical approaches to functionalize the surface with desired agents [18,19,20] or can be used to immobilize active agents within the forming polymeric coating [21,22,23]. Even after fifteen years of discovery, this approach is still attracting a lot of attention, and the number of described systems and their potential applications is constantly growing [14,24,25,26,27].

Further development of the conception has shifted attention from commercially-available dopamine to other catechol systems and dopamine derivatives, giving rise to polydopamine-like coatings. This approach relies on dopamine derivatives that are made from dopamine or other catecholic compounds by synthetic approaches with a subsequent application for surface modification [28,29,30,31]. This strategy makes it possible to change the sequence of functionalization: firstly, functional monomers can be synthesized by conventional organic synthesis, in which case the coating is applied on the substrate’s surface. Separation of the “organic” and the “colloidal” stages not only simplifies the characterization and purification but also makes it possible to increase the ratio between the applied and successfully immobilized motifs. Despite its potential benefits, this approach has received much less attention. Despite this, its potential will be further revealed by other researchers [25,32,33].

Here we highlight the potency of pre-modified dopamine application in the creation of functional nanocomposites. We chose magnetite nanoparticles as a substrate due to their unique magnetic properties and excellent biocompatibility, and successfully modified them to produce model bioactive hybrid materials. We synthesized several dopamine conjugates and compared the effectiveness of modification using these agents on three types of magnetite nanoparticles. The dependencies of the modification results on the synthetic conditions and bioactivity of the synthesized composites on model systems are presented.

## 2. Results

### 2.1. Magnetic Nanoparticles

Initial efforts were aimed at finding an optimal nanoparticle to be used for further modifications. Among the great variety of the known magnetic nanosystems, we decided to consider three types of magnetite nanoparticles in order to define the best and most convenient one. The first system was magnetite hydrosol (Ferria), which has been described in our previous works [34,35]. This system was a hydrocolloid of octahedron-shaped magnetite nanoparticles with a mean diameter of 10 nm dispersed in deionized water with excellent colloidal stability at a neutral pH level without any added surface-modifying agents or peptization agents in its composition. Due to the non-stochiometric ratio of Fe(II) and Fe(III) ions during its synthesis, the surface of the nanoparticles is enriched with Fe(II)–OH groups that shift the isoelectric point of the material from magnetite’s typical pH of 6.8 [36] to pH 8.2, resulting in the highly positive zeta potential of the system valued +32 mV at neutral pH level [35,37].

**Figure 1:**
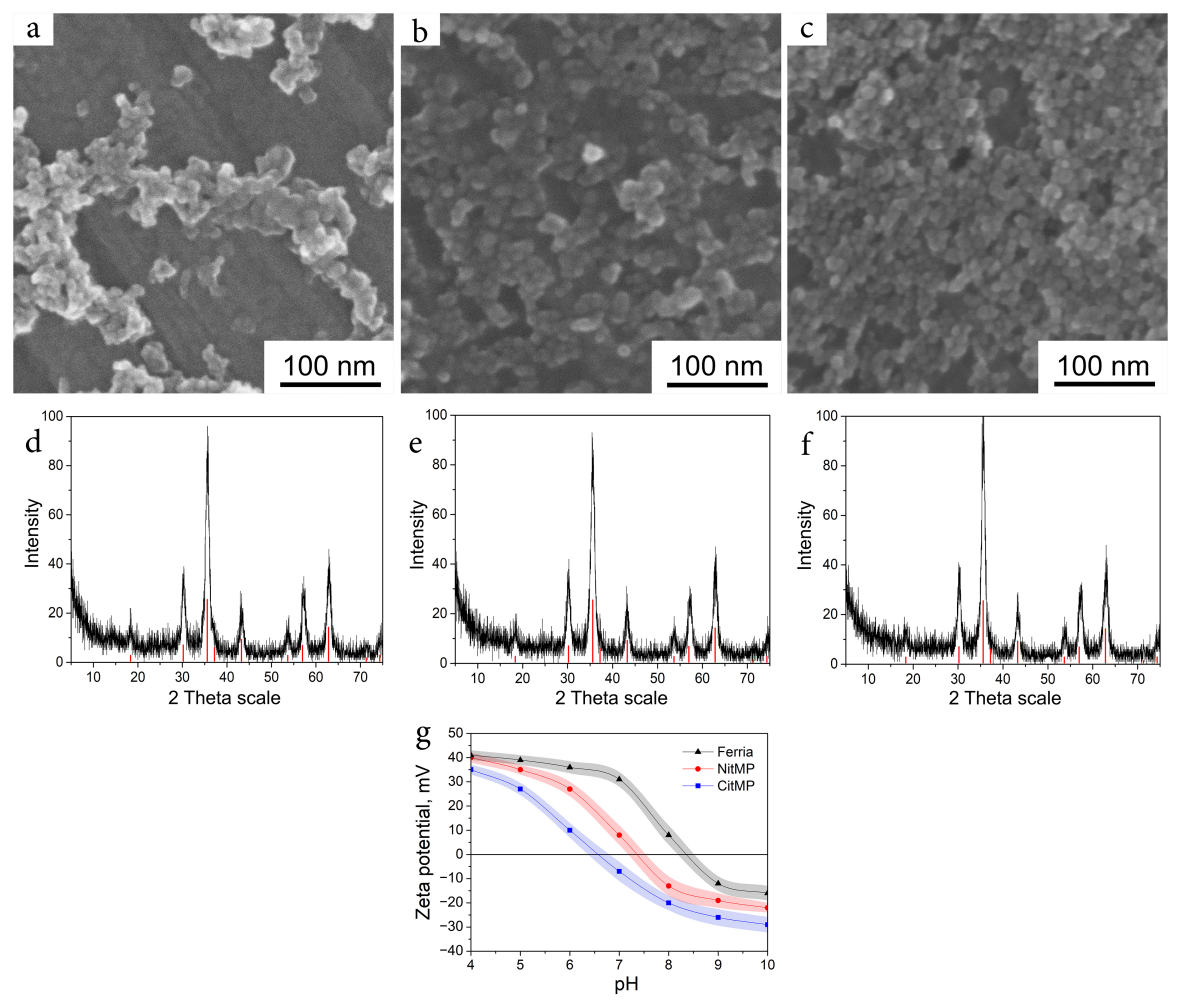
Magnetite nanoparticles in this study. SEM image and XRD pattern of Ferria (**a**,**d**), NitMP (**b**,**e**), and CitNitMP (**c**,**f**). (**g**): Zeta potential at various pH levels for all synthesized hydrocolloids.

Two more magnetite hydrosols were used to evaluate the effect of surface chemistry on the effectiveness of biomimetic surface modification. The second tested system was the well-known and widely applied citrate-capped magnetite hydrosol (CitMP) nanoparticles. This type of magnetite hydrocolloid is based on the ability of citric anions to coordinate on the surface of magnetite nanoparticles by two carboxylic and one hydroxyl group, resulting in highly charged and stable hydrosols [38,39,40,41]. The third system was another typical and well-described magnetic colloid that consisted of magnetite hydrosol which was stabilized by nitric acid (NitMP). Nitric acid is a well-known peptization agent widely applied in sol-gel chemistry to charge surfaces of nanoparticles by increasing H${}^{+}$ concentration near their surface [42,43]. All named systems have been synthesized by co-precipitation procedures; thus, all had near-spherical shapes as was seen from SEM images (Figure 1a–c). All three materials had almost identical XRD patterns (Figure 1d–f) that demonstrated the main peak at 35.58${}^{{}^{\circ}}$ attributed to the crystalline plane with Miller indices of (311) as well as other distinctive peaks at 18.36${}^{{}^{\circ}}$ (111), 30.10${}^{{}^{\circ}}$ (220), 43.25${}^{{}^{\circ}}$ (400), 53.66${}^{{}^{\circ}}$ (422), 57.20${}^{{}^{\circ}}$ (511), 62.82${}^{{}^{\circ}}$ (440), 71.28${}^{{}^{\circ}}$ (620), and 74.33${}^{{}^{\circ}}$ (533) typical for magnetite crystalline phase (RRUFF No. R061111). The crystallite sizes were calculated by the Scherrer equation and were valued between 10 and 11 nm, proving the propinquity of the synthesized nanoparticles parameters. The main difference between the named systems was the approach to their stabilization that resulted in differences in their surface chemistry and hydrodynamic parameters (Table 1). The most striking difference between used systems can be seen in their isoelectric points valued at 6.3 to 7.3 and 8.2 for CitMP, NitMP, and Ferria, respectively (Table 1 and Figure 1g). This difference originated from the nature of their surface charge: while for NitMP and Ferria the charge originated from Fe–OH hydroxyl groups on the surface, for CitMP it was also affected by carboxylic groups of citrate residues on the surface of nanoparticles that shift the isoelectric point to a lower pH.

The comparative characteristics of the used systems can be seen in Table 1.

### 2.2. Biomimetic Coating of the Nanoparticles

**Figure 2:**
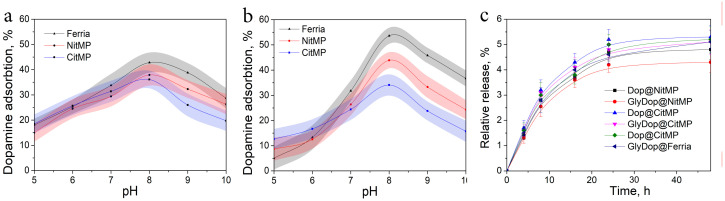
Adsorption of dopamine (**a**) and GlyDop (**b**) on nanoparticles as a function of pH level. (**c**) Stability of the coatings in PBS.

The surface of the synthesized NPs was modified via biomimetic polymerization of dopamine derivatives. In the initial experiments, the applicability of dopamine derivatives for this process was evaluated. For this purpose we firstly compared the effectiveness of nanoparticle modification by dopamine and dopamine conjugate using glycine (GlyDop) at various pH levels. The coating efficiency for both catechols showed nonlinear dependence on pH level, with a local maximum near pH 8, which correlates with previously published data [44,45]. The effectiveness of coating the used nanoparticle substrates with unmodified dopamine was nearly identical at acidic pH levels and showed a minor difference in the basic pH region, resulting in up to 42.8%, 37.94% and 36.1% wt. Dop absorption for Ferria, NitMP, and CitMP, respectively (Figure 2a). When coating with GlyDop, more pronounced dependence on the NPs surface charge and chemistry was observed (Figure 2b), with much higher efficiency for Ferria NPs reaching 53.6% at pH 8.0, while for NitMP, and CitMP these values were 43.9% and 34.1%, respectively. To evaluate the stability of the coatings, composite materials were incubated in PBS buffer and leached components were detected by measuring the optical density of supernatants. The experiments demonstrated good stability of the materials with only a minor degree of release, around 4–5% for all the tested systems (Figure 2c).

**Figure 3:**
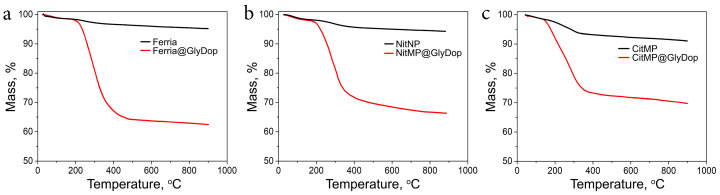
TGA curves of Ferria (**a**), NitMP (**b**), and CitMP (**c**), and the corresponding composites with GlyDop.

The compositions of the resulting materials were additionally evaluated by TGA measurements, which demonstrated the mass fraction of polydopamine coating in GlyDop@Ferria, GlyDop@NitMP, and GlyDop@CitMP at levels of 32%, 28% and 21% wt., respectively (Figure 3a–c), corresponding to 10.5%, 9.3% and 7% wt. of glycine fragment in their composition, respectively.

**Figure 4:**
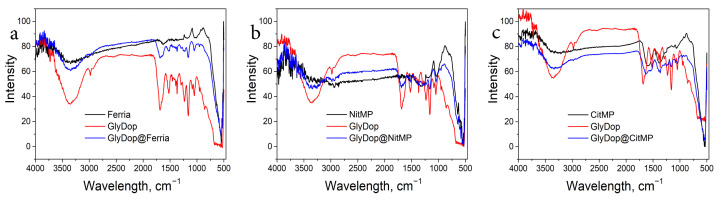
FTIR spectra of Ferria (**a**), NitMP (**b**), and CitMP (**c**), and the corresponding composites with GlyDop.

To confirm the successful modification of the organo-inorganic composites, the materials were characterized with FTIR spectroscopy in order to determine the characteristic functional groups. The measurements were carried out for all three types of MNPs and GlyDop@MNPs composites. The results are presented at Figure 4. All MNPs are characterized by strong bands near 550 cm${}^{-1}$ with a shoulder at 630 cm${}^{-1}$ that correspond to Fe–O vibration and a broad band at 3000–3300 cm${}^{-1}$ due to –OH groups from adsorbed water molecules and Fe–OH groups. [46]. For spectra of hybrid materials, the broad band at 3500–3300 cm${}^{-1}$ is much more pronounced due to characteristic stretching vibrations of –OH and –N–H groups. A strong band at approximately 1660 cm${}^{-1}$ can be attributed to an Amide I band which overlays –C=C stretching vibrations in aromatic rings located near 1600 cm${}^{-1}$. At 1525 cm${}^{-1}$ Amide II band can be seen accompanied by the peak at 1047 cm${}^{-1}$, corresponding to C=O vibrations of amide groups [47]. Presence of the above-mentioned peaks indicates the composition of the hybrid materials.

**Figure 5:**
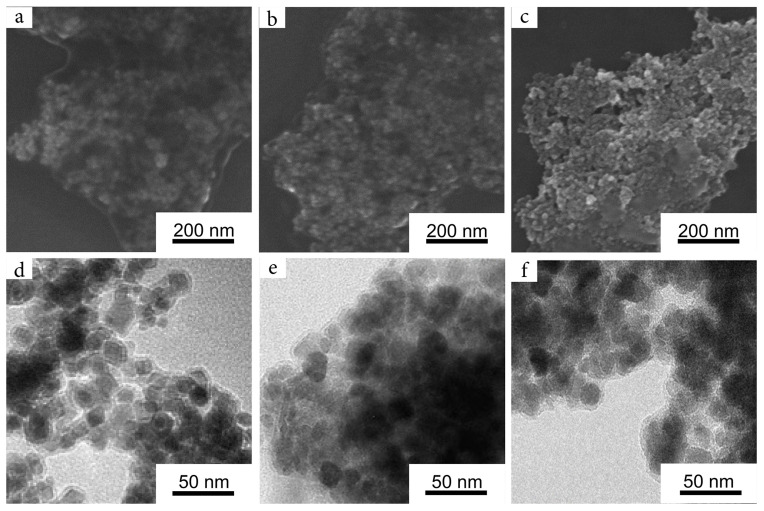
SEM and TEM images of GlyDop@Ferria (**a**,**d**), GlyDop@NitMP (**b**,**e**), and GlyDop@CitMP (**c**,**f**), respectively.

Visualization of the composites with SEM and TEM showed that the obtained materials maintained near-spherical morphology (Figure 5). It can be seen that composite materials consist of individual nanoparticles coated with an organic layer with a thickness of 2–3 nm when visualized in the dried form. This observation was partially confirmed by DLS measurements, which valued the hydrodynamic radii of the particles at 48 nm, 45 nm, and 40 nm for GlyDop@CitMP, GlyDop@NitMP, and GlyDop@Ferria, respectively. The particles had positive zeta potential due to amino-groups on their surface originating from glycine residues, and were had values close to each, at +25 mV, +27 mV, and +26 mV, respectively.

### 2.3. Bioactivity of the Dopamine-Coated NPs

**Figure 6:**
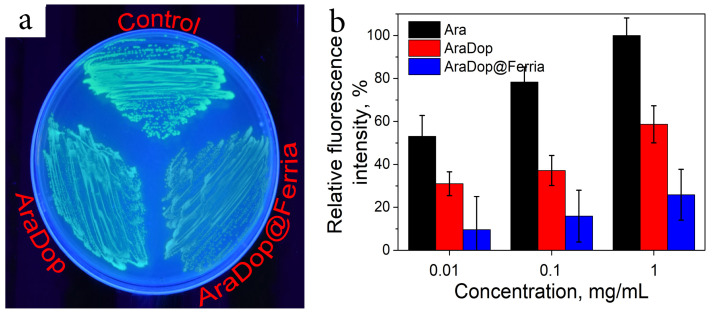
Bioactivity of AraDop@Ferria. (**a**) Visual appearance of fluorescent *E. coli* Nova Blue pBad; (**b**) Fluorescent signal in response to various arabinose species.

Biomimetic modification of nanoparticles offers a quick and convenient strategy for surface engineering and may be used to alternate the bioactivity of nanoparticle systems. To evaluate this possibility, two systems were prepared and their bioactivity was tested on bacteria models. In the first model, MNPs were modified with the conjugate of dopamine and arabinose (AraDop). The mass fraction of the organic component was estimated at the level of 11% wt according to UV studies, the produced particles had a hydrodynamic radius of 40 nm, and zeta potential valued at +18 mV. The bioavailability of the material was evaluated on *E. coli* Nova Blue pBad strain which was genetically designed to produce Green Fluorescent Protein (GFP) when arabinose is added to the nutrient media and compared to AraDop conjugate and free arabinose. The concentration of GFP was measured as a fluorescence intensity at 532 nm, thus allowing for evaluation of the bioactivity of the systems. The results are presented in Figure 6. All the tested systems caused the appearance of a fluorescent signal which showed a direct dependence on arabinose species concentrations. The conjugated form of arabinose caused approximately a two and four times lower biological response for AraDop and AraDop@Ferria compared to free Ara on all the tested concentrations in the range from 0.01 to 1 mg/mL.

**Figure 7:**
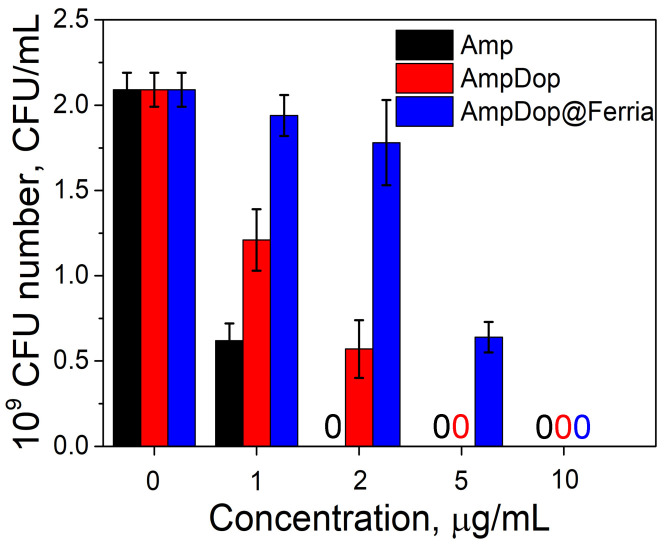
Antibiotic activity of AmpDop@Ferria.

As the second model system, we have selected Ferria NPs modified with ampicillin-dopamine conjugate AmpDop@Ferria. The mass fraction of the organic component was estimated at the level of 15% wt. according to UV studies, the produced particles had a hydrodynamic radius of 82 nm, and zeta potential valued at +16 mV. Such a large hydrodynamic radius may be attributed to the relatively high hydrophobicity of the conjugate and partial aggregation of the NPs in water media. Antibacterial activity of the conjugate was evaluated on ampicillin-sensitive *E. coli* strain ATCC 25922 (Figure 7). Based on the results of the experiment, we can conclude that the antibacterial activity of conjugated ampicillin decreased compared to its free form. In the experiment with the conjugate, the minimum inhibitory concentrations (MIC) were found at 5 and 10 μg/mL for AmpDop and AmpDop@Ferria, respectively, while for free ampicillin MIC was found at the level of 2 μg/mL, while control experiments showed absence of any antibacterial activity for dopamine or Ferria.

## 3. Discussion

**Figure 8:**
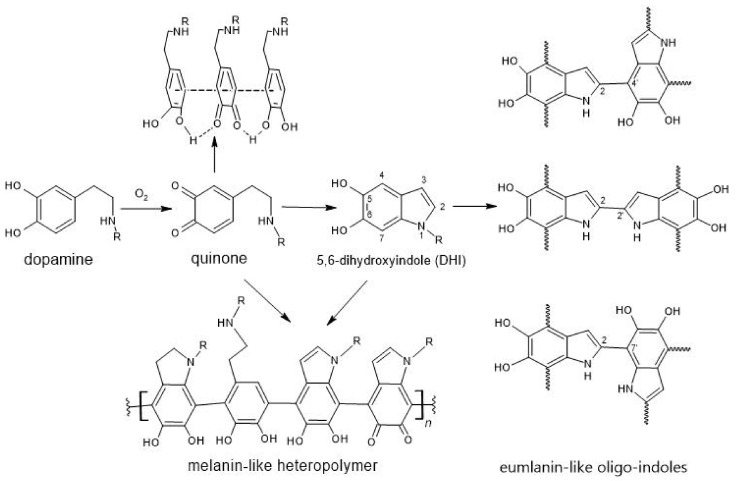
Schematic representation of dopamine polymerization.

Here we showed the potency of the biomimetic strategy of nanoparticles surface modification by dopamine derivatives in-situ polymerization under mild conditions for the production of bioactive nanocomposites. This strategy relies on oxidative cyclization of dopamine under mild conditions resulting in its polymerization in the manner shown in Figure 8. The exact mechanism behind the process remains unknown, but its main features are broadly discussed in the literature [12,48,49]. In the first step, dopamine is oxidized by dissolved oxygen forming a quinone intermediate. The formed quinone can undergo intramolecular cyclization leading to 5,6-dihydroxyindole (DHI), the second important molecule. It is thought that these two compounds and initial dopamine can interact in various ways, including covalent inter-molecular bonding resulting in melanin- and eumelanin-like structures. Another possible way of interaction includes non-covalent self-assembly through hydrophobic interactions, hydrogen bonds or π–π stacking [12,13]. The overall PDA layer formation process consists of two acts: dopamine polymerization and its adsorption to the surface.

It is known that bidentate catechols such as dopamine and its derivatives effectively bind to iron oxide primary through dissociated catechol groups. This process converts under-coordinated Fe–O surface sites back to an octahedral bulk-like lattice structure. The effectiveness of this process is dependent on the surface chemistry. Generally, the thickness of the formed surface correlates with the hydrophobicity of the surface and its charge in the reaction conditions [13]. It is known that a higher negative charge of the surface inhibits the rate of adsorption of the dissociated catechol groups, leading to lower rates of nucleation and deposition of the organic polymer. This fact had beeen reflected in our study. We used three types of iron oxide with close hydrodynamic radius but different surface properties, namely magnetite nanoparticles coated with citric acid (CitMP), nitric acid (NitMP), and iron hydroxide (Ferria). Comparative deposition of PDA layers under the same conditions showed lower effectiveness in the order Ferria>NItMP>CitMP, following the decline in the substrate isoelectric point and higher negative charge of the surface.

As was shown in the literature and mentioned earlier, the rate of PDA deposition is dependent on several processes: adsorption of catechols, formation rates of quinone and 5,6-dihydroindole, and the rates of melanin- and eumelanin-like polymer generation and their nucleation. All of these processes are occurring through protonation and deprotonation, so the pH level plays an important role in the PDA layer formation process. It is known that the rate of quinone and 5,6-dihydroindole formation accelerates at a higher pH level. At the same time, it was found that high pH levels lower the effectiveness of PDA deposition due to the dissociation of phenolic hydroxyls at their pK${}_{a}$ point near pH 9.0–9.5. Since the process of catechols adsorption to the surface is the primary step in the deposition of the polydopamine layer, dissociation of catechols at higher pH becomes a dominant effect that favors the dissolution of the polymer rather than its adsorption to the surface [50]. This phenomenon was observed in our study and as the effectiveness of the modification for all the tested systems was maximal at pH 8.0, higher pH levels led to a lower level of organic component in hybrid NPs.

It was demonstrated that biomimetic modification of NPs surfaces with dopamine derivatives can be used for the synthesis of bioactive composite materials in a fast and convenient way. Preliminary synthesis of dopamine conjugates with subsequent deposition of PDA-like layers gives better control over the whole process and allows separation of the “organic” stage from the “colloidal” one. This approach showed excellent loading capacities of the NPs with bioactive molecules which reached up to 10.5% wt. in the case of GlyDop due to volumetric deposition of bioactive molecules in the polymer layer. In comparison, post-deposition surface modification is typically limited by modification densities on the level of 0.01–0.5 molecules per square nanometer resulting in loadings up to 1–3% wt. [51,52,53]. In addition, the application of dopamine conjugates instead of intact dopamine potentially allows production of more complex systems by using several derivatives simultaneously during the polymerization stage, leading to multi-functional systems.

**Figure 9:**
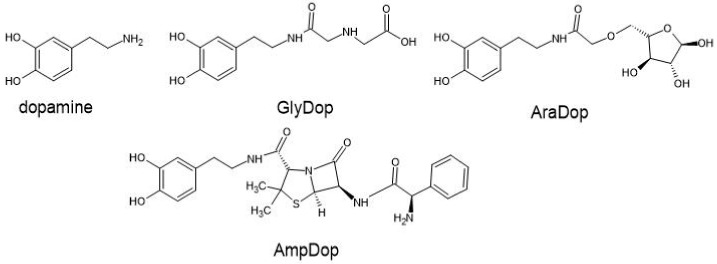
Structures of dopamine and dopamine derivatives in this study.

The bioactivity of the magnetic nanoparticles can be modulated by the attachment of dopamine conjugates. In this study, we demonstrated that Ferria can be made either biofriendly or biocidal by modification with AraDop or AmpDop conjugate, respectively. Conjugation of arabinose or ampicillin to dopamine led to an approximately two-fold decrease in their activities in *E. coli*. The conjugates AraDop@Ferria and AmpDop@Ferria were even less effective with approximately four and five times lower activity compared to intact molecules at maximal tested concentrations. To understand this fact, it is important to understand the mechanisms of cell interactions with bioactive molecules. It is known that L-arabinose uptake in *E. coli* is mediated via the arabinose transporters AraE and AraFGH, and regulated by AraC protein that actively represses transcription of the ara${}_{BAD}$ operon in the absence of the substrate. The interaction of AraC with arabinose occurs in the sugar-binding pocket though protein main chain residues Leu${}^{9}$, Leu${}^{10}$, Gly${}^{12}$, Tyr${}^{13}$, and Phe${}^{15}$ in the NH2-terminal arm and requires arabinose to be in its pyranose (six-membered ring) form [54]. In AraDop conjugate arabinose is presented in its five-membered ring furanose form (see Figure 9 AraDop conjugate structure), so in order to be active the conjugate must firstly be hydrolyzed, and then arabinose can be converted into its pyranose form by the corresponding mutase. It is possible that slow rates of hydrolysis and lower affinities of AraE and AraFGH to AraDop conjugate determine the two-fold decrease in its activity.

In its turn, the mechanism of ampicillin antibacterial activity is mediated through its interaction with penicillin-binding proteins (PBPs). PBPs are transpeptidases or carboxypeptidases involved in the synthesis of peptidoglycan, which is the major component of bacterial cell walls. Ampicillin and other β-lactams act as irreversible inhibitors by forming covalent complexes, with the active site of PBPs effectively preventing their further activity [55]. The interaction of PBPs and ampicillin is mediated through its carboxyl group, which was used to prepare AmpDop conjugate through an amide bond; thus, this bond must be hydrolyzed to release the active molecule (see Figure 9 AraDop conjugate structure).

Taking into account the above-mentioned issues, it is not surprising that AraDop@Ferria and AmpDop@Ferria had even lower activities compared to free molecules. Due to the high stability of the synthesized coatings, the spontaneous release of AraDop and AmpDop or corresponding bioactive residues is almost absent, so implementation of the bioactivity is possible only after the internalization of nanoparticles within bacteria. It is known from the literature that nanoparticles can be taken up by *E. coli* with a varied efficiency depending on various factors. The mechanisms of these processes are unknown. It was shown that the uptake efficiency can be modulated by NPs surface modification or by addition of various additives such as tissue homogenate S9 fraction containing cytosol and microsomes [56,57,58,59]. The synthesized composites showed moderate activity compared to free bioactive molecules, but the obtained results can be considered positive for un-optimized systems. Moreover, the magnetic properties of the composites can also be beneficial for the localization of such materials and improvement of their bioactivity.

## 4. Conclusions

Here, we demonstrated the concept of nanoparticle surface modification based on pre-synthesized dopamine conjugates. Magnetic nanoparticles were modified with dopamine conjugates under mild conditions, leading to organo-inorganic composites with a high rate of the organic component. Application of dopamine conjugates enabled achievement of highly active molecules, loading up to 10.5% wt. The synthesized systems demonstrated bioactivity inherent to the used dopamine conjugates. The contingency of the modification process and bioactivity of the synthesized materials proved the potency of the proposed strategy, which will be further evaluated in the upcoming works.

## 5. Materials and Methods

*Chemicals:* All reagents and solvents were obtained or distilled according to standard procedures. The reagents used for experiments were purchased from Sigma-Aldrich Co. and used as received unless otherwise noted. *N,N*-dimethylformamide (DMF), acetonitrile, 1,4-dioxane, and tetrahydrofuran (THF) were distilled from phosphorus pentoxide under argon, and solvents were stored under argon.

*Bacterial Strains: E. coli* strain ATCC 25922 and *E. coli* XL1 Blue carrying plasmid pBADcycle3-mutant strains were grown at 37 ${}^{{}^{\circ}}$C in lysogeny broth (LB) moderately supplemented with 12.5 μg/mL of tetracycline (PanReac AppliChem, Barcelona, Spain).

*Magnetite hydrosol (**Ferria**):* Stable magnetite hydrosol was prepared by the procedure described earlier [34,60]. Briefly, 2.5 g FeCl${}_{2}\cdot$4H${}_{2}$O and 5 g FeCl${}_{3}\cdot$6H${}_{2}$O were dissolved in 100 mL of deionized water. Then, 11 mL of 30% NH${}_{4}$OH was added under constant stirring (500 rpm) at room temperature. The formed magnetite precipitate was magnetically separated and washed with deionized water until a neutral pH was obtained. The washed black precipitate was mixed with 100 mL of deionized water and subjected to ultrasonic treatment (37 kHz, 110 W) under constant stirring (300 rpm). The resulting mass concentration of the magnetite hydrosol was 2%.

*Nitric-acid-stabilized magnetite (**NitMP**):* Nanoparticles were synthesized according to the procedure from [61]. Briefly, 5.9 g of FeCl${}_{3}\cdot$6H${}_{2}$O and 2.15 g of FeCl${}_{2}\cdot$4H${}_{2}$O were dissolved in 100 mL of degassed deionized water. 12.5 mL of 30% NH${}_{4}$OH was quickly added. The solution was heated to 85 ${}^{{}^{\circ}}$C and stirred for 1 h. Then the formed magnetic particles were magnetically separated and washed sequentially with 2 M HNO${}_{3}$ and thrice with deionized water. The resulting suspension was diluted with deionized water to the final mass concentration of 2% wt.

*Citric-acid-stabilized magnetite (**CitMP**):* Nanoparticles were synthesized as described in [62]. Briefly, FeCl${}_{2}\cdot$4H${}_{2}$O (2.5 g) and FeCl${}_{3}\cdot$6H${}_{2}$O (4.0 g) were dissolved in 180 mL of distilled water under nitrogen gas. 50 mL of sodium hydroxide was dropwise added to the reaction mixture and kept for 10 min at 65 ${}^{{}^{\circ}}$C under continuous vigorous stirring. Then, 150 mL of 5% citric acid solution was added to the reaction mixture, which was then stirred for 10 min (65 ${}^{{}^{\circ}}$C). CitMP was collected with a permanent magnet and thoroughly rinsed four times with distilled water. The resulting suspension was diluted with deionized water to the final mass concentration of 2% wt.

*N-Chloroacetyl-3,4-dihydroxyphenethylamine:* To an aqueous solution of dopamine hydrochloride (1.89 g, 10 mmol) was added, under nitrogen, 25 mL of 2.0 N sodium hydroxide and then an ethereal solution of chloroacetic anhydride (1.71 g, 15.2 mmol). Vigorous stirring was continued for 0.5 hr. More sodium hydroxide (10 mL, 2 equiv) and 1.71 g of chloroacetic anhydride were added under stirring. The reaction mixture was continuously stirred for 1 hr more. The reaction mixture was neutralized to pH 6 by the addition of hydrochloric acid and extracted with ethyl acetate four times. The combined extracts left an oily residue, 2.4 g, which was chromatographed on a column of 45 g of silica gel. The column was eluted with chloroform containing 4% methanol, and fractions of 7 mL were collected. Fractions 30–50 contained pure N-chloroacetyl-3,4-dihydroxyphenethylamine which was recrystallized from chloroform to yield colorless crystalline granules (1.52 g, 67% yield), mp 108–109 ${}^{{}^{\circ}}$C. HRMS for C${}_{10}$H${}_{12}$ClNO${}_{3}$ [M+H]${}^{+}$ Calc.: 231.0476, found: 231.0462

*(2-((3,4-dihydroxyphenethyl)amino)-2-oxoethyl)glycine (**GlyDop**):* To a solution of N-Chloroacetyl-3,4-dihydroxyphenethylamine (1 g, 4.4 mmol) in 1,4-dioxane (20 mL) was added, under nitrogen, 25 mL of 2.0 N sodium hydroxide and then p-tosylglycine (1 g, 4.4 mmol). Vigorous stirring was continued for 3 hr. The reaction mixture was neutralized to pH 1 by the addition of hydrochloric acid and extracted with ethyl acetate four times. The organic extracts were combined and dried over MgSO${}_{4}$, filtered and evaporated in vacuo. The crude residue was purified by column chromatography (silica, CHCl${}_{3}$-MeOH (5:1)) to give the target product (820 mg, 70% yield).

${}^{1}$H NMR (600 MHz, MeOD) σ 7.87–7.84 (2H, m, ArH), 7.51–7.46 (1H, m, ArH), 3.50 (2H, t, J = 7.3 Hz, CH${}_{2}$N), 3.46 (2H, s, CH${}_{2}$), 3.22 (2H, s, CH${}_{2}$), 2.74 (2H, t, J = 7.3 Hz, CH${}_{2}$Ar); ${}^{13}$C NMR (150 MHz, MeOD) σ 173.1, 170.7, 145.6, 144.5, 131.5, 122.8, 116.4, 52.9, 50.7, 40.3, 39.4 HRMS for C${}_{12}$H${}_{16}$N${}_{2}$O${}_{5}$ [M+H]${}^{+}$ Calc.: 269.1093, found: 269.1087

*Conjugate of dopamine with arabinose (**AraDop**):* A suspension of D-arabinose (0.5 g, 3.33 mmol) and potassium carbonate (0.69 g, 5 mmol) in anhydrous acetonitrile (15 mL) was stirred at room temperature under N${}_{2}$ atmosphere for 15 min. Then N-chloroacetyl-3,4-dihydroxyphenethylamine (0.76 g, 3.33 mmol) was added, and stirring was prolonged for 12 h. The reaction was monitored by TLC using petroleum hexane/ethyl acetate 1:2 (*v*/*v*) as the eluent. The solvent was removed under reduced pressure and the residue purified by flash silica gel column chromatography using hexane/ethyl acetate 1:2 (*v*/*v*) as the eluent to yield conjugate (540 mg, 87% yield). Mp 112–114 ${}^{{}^{\circ}}$C;

${}^{1}$H NMR (600 MHz, MeOD) σ 7.85–7.82 (2H, m, ArH), 7.50–7.46 (1H, m, ArH), 5.85–5.83 (1H, m, H-1′), 4.59–4.56 (1H, m, H-4′), 4.50–4.48 (1H, m, H-3′), 4.42–4.40 (1H, m, 3′-OH), 4.32–4.29 (1H, m, 2′-OH), 3.91–3.88 (1H, m, H-2′), 3.59–3.56 (1H, m, 5′-CH${}_{2}$), 3.55 (2H, s, CH${}_{2}$), 3.47 (2H, t, J = 7.3 Hz, CH${}_{2}$N), 3.36–3.34 (1H, m, 5′-CH${}_{2}$), 2.75 (2H, t, J = 7.3 Hz, CH${}_{2}$Ar); ${}^{13}$C NMR (150 MHz, MeOD) σ 168.6, 145.3, 144.7, 131.5, 122.8, 116.4, 115.9, 102.7, 84.0, 76.7, 76.5, 72.0, 68.8, 40.6, 39.4 HRMS for C${}_{15}$H${}_{21}$NO${}_{8}$ [M+H]${}^{+}$ Calc.: 344.1301, found: 344.1324

*Conjugate of dopamine with ampicillin (**AmpDop**):* A solution of ampicillin (0.5 g, 1.43 mmol) and CDI (0.42 g, 2.58 mmol) in anhydrous DMF (15 mL) was stirred at room temperature under N${}_{2}$ atmosphere for 15 min. Then, dopamine (0.27 g, 1.43 mmol) was added, and stirring was prolonged for 12 h. The reaction was monitored by TLC using petroleum ether/ethyl acetate 2:8 (*v*/*v*) as the eluent. The solvent was removed under reduced pressure and the residue was purified by flash silica gel column chromatography using petroleum ether/ethyl acetate 2:8 (*v*/*v*) as the eluent to yield conjugate (600 mg, 87% yield). Mp 99–102 ${}^{{}^{\circ}}$C;

${}^{1}$H NMR (600 MHz, MeOD) σ 8.72 (2H, s, NH${}_{2}$), 7.85–7.82 (2H, m, ArH), 7.50–7.46 (1H, m, ArH), 7.39–7.36 (2H, m, ArH), 7.34–7.32 (1H, m, ArH), 7.30–7.27 (2H, m, ArH), 5.22–5.19 (1H, m, CH), 4.87–4.84 (1H, m, CH), 4.82–4.78 (1H, m, CH), 3.47 (2H, t, J = 7.3 Hz, CH${}_{2}$N), 2.75 (2H, t, J = 7.3 Hz, CH${}_{2}$Ar), 1.58 (3H, s, CH${}_{3}$), 1.53 (3H, s, CH${}_{3}$); ${}^{13}$C NMR (150 MHz, MeOD) σ 174.5, 171.0, 169.2, 145.6, 144.5, 133.6, 131.5, 129.6, 129.2, 127.6, 122.8, 116.4, 115.9, 87.0, 71.8, 65.0, 60.7, 57.2, 40.6, 39.4, 29.4, 29.2 HRMS for C${}_{24}$H${}_{28}$N${}_{4}$O${}_{5}$S [M+H]${}^{+}$ Calc.: 485.1814, found: 485.1822

*PDA layer formation on MNPs:* For PDA layer formation experiments, 100 μL of magnetite NPs was mixed with a 100 μL of 2% dopamine derivative solution in methanol and 200 μL of PBS or acetate buffer with the desired pH was added. The system was incubated for 24 h, magnetically separated, and washed six times with 1 mL of a buffer. The collected liquid fractions were combined and analyzed with UV spectroscopy. The magnetic material was washed with water and dried.

*Stability of PDA layer:* 1 mg of the composite was dispersed in 1 mL of PBS and incubated at 20 ${}^{c}irc$C on rotator. After specific time periods magnetic nanoparticles were separated by centrifugation at 15000 G, and aliquotes of supernatant were collected and analysed by UV spectroscopy.

*Antibiotic activity testing:* The strain of *E. coli* ATCC 25922, which is sensitive to ampicillin, was used for the experiment. 5 μL of bacteria suspension was added to test tubes with 1 mL LB of nutrient medium and cultured for 24 h. After that, the tested substance was added and the culture was grown for another 24 h in 1 mL of LB culture medium for 24 h at 37 ${}^{{}^{\circ}}$C in a shaker incubator at 250 rpm and optical density was measured.

*Bacteria biofluorescence experiments:* 5 μL of *E. coli* XL1 Blue suspension was added to test tubes with 1 mL LB of nutrient medium and cultured for 24 h. After that, the tested substance was added and the culture was grown for another 24 h in 1 mL of LB culture medium for 24 h at 37 ${}^{{}^{\circ}}$C in a shaker incubator at 250 rpm and fluorescence was measured.

## Figures and Tables

**Table 1:** Magnetite hydrosols used in this study.

Name	pH of the Colloid	Surface Groups	Crystallite Diameter, nm	Hydrodynamic Radius, nm	Isoelectric Point, pH	ζ Potential at pH 7, mV
CitMP	4.6	–COOH	10.47	40	6.3	−7
NitMP	2.3	Fe–OH${}_{2}$${}^{+}$	10.74	38	7.3	+8
Ferria	6.8	Fe(II)–OH${}_{2}$${}^{+}$	10.31	33	8.2	+32

## Data Availability

Not applicable.
